# Protein Kinase Inhibitor Peptide as a Tool to Specifically Inhibit Protein Kinase A

**DOI:** 10.3389/fphys.2020.574030

**Published:** 2020-11-25

**Authors:** Chong Liu, Ping Ke, Jingjing Zhang, Xiaoying Zhang, Xiongwen Chen

**Affiliations:** ^1^ Department of Pharmacology, Second Military Medical University, Shanghai, China; ^2^ Cardiovascular Research Center, Temple University School of Medicine, Philadelphia, PA, United States

**Keywords:** protein kinase inhibitor peptide, protein kinase A, cyclic adenosine monophosphate, H89, protein kinase A inhibition

## Abstract

The protein kinase enzyme family plays a pivotal role in almost every aspect of cellular function, including cellular metabolism, division, proliferation, transcription, movement, and survival. Protein kinase A (PKA), whose activation is triggered by cyclic adenosine monophosphate (cAMP), is widely distributed in various systems and tissues throughout the body and highly related to pathogenesis and progression of various kinds of diseases. The inhibition of PKA activation is essential for the study of PKA functions. Protein kinase inhibitor peptide (PKI) is a potent, heat-stable, and specific PKA inhibitor. It has been demonstrated that PKI can block PKA-mediated phosphorylase activation. Since then, researchers have a lot of knowledge about PKI. PKI is considered to be the most effective and specific method to inhibit PKA and is widely used in related research. In this review, we will first introduce the knowledge on the activation of PKA and mechanisms related on the inhibitory effects of PKI on PKA. Then, we will compare PKI-mediated PKA inhibition vs. several popular methods of PKA inhibition.

## Introduction

The protein kinase enzyme family plays a pivotal role in almost every aspect of cellular function, including cellular metabolism, division, proliferation, transcription, movement, and survival (Manning et al., 2002). Protein kinases function by phosphorylating proteins, which is balanced with dephosphorylation by phosphoprotein phosphatases, making phosphorylation-dephosphorylation an effective regulatory process (Alonso et al., 2004; Roskoski, 2015).

Protein kinase A (PKA) was the first protein kinase to be discovered. It was identified in skeletal muscle by Edmond H. Fisher and Edwin G. Krebs when they found that glycogen phosphorylase was activated following its phosphorylation by protein kinase, which was later termed “phosphorylase kinase” and now “protein kinase A” (Krebs and Fischer, 1956; Krebs et al., 1958). Almost at the same time, Sutherland et al. (Rall and Sutherland, 1958; Sutherland and Rall, 1958) demonstrated that cyclic adenosine monophosphate (cAMP), a heat-stable compound initially characterized in 1957 by Cook and the colleagues, led to the activation of PKA. As such, PKA is also termed “cAMP-dependent protein kinase.”

As a prototypical serine/threonine kinase, PKA is distributed in all systems and tissues throughout the body and related to pathogenesis and progression of various diseases. Numerous studies have focused on the function, regulation, and pathological roles of this signaling pathway. Manipulation of this signaling pathway has been explored to treat several diseases such as cardiovascular diseases, Alzheimer disease, Parkinson’s disease, ischemia, and diabetes. (Kleppe et al., 2011; Dema et al., 2015; Sapio et al., 2017; Wild and Dell’Acqua, 2018).

## cAMP/PKA Signaling

The discovery of cAMP-mediated activation of PKA led to the “Second Messenger Hypothesis” in cellular signaling. It is described as that extracellular ligands, such as a certain kind of hormones like glucagon and epinephrine acted on membrane receptors and cause the generation of intracellular second messengers like cAMP, which in turn activated a kind of protein kinase and lead to the activation of cellular processes like glycogenolysis (Rall and Sutherland, 1958; Sutherland and Rall, 1958). cAMP is the first described second messenger, which generated by activated adenylyl cylase (Sutherland and Robison, 1966).

**Figure 1:**
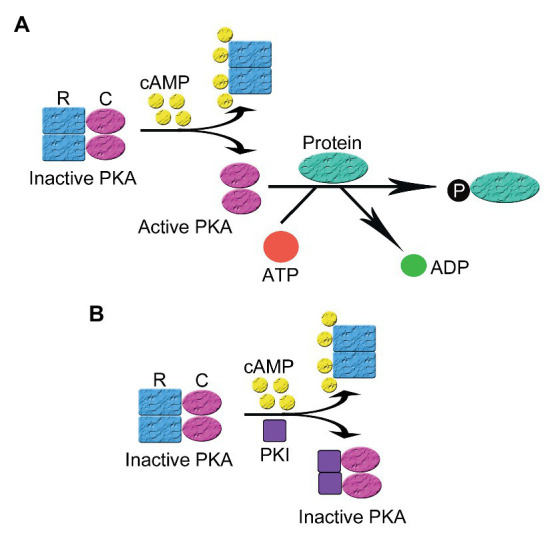
The activation and inhibition of cyclic adenosine monophosphate (cAMP)-dependent protein kinase A (PKA). **(A)** In the absence of cAMP, PKA is an inactive tetrameric holoenzyme consisted of two regulatory (R) and two catalytic subunits (C). In the presence of cAMP, each R subunit binds to two molecules of cAMP at separate allosteric binding sites, leading to the decrease of the affinity between the R and the C subunits and the separation of the holoenzyme of PKA into a regulatory subunit dimer and two monomeric catalytic subunits. The released C subunits becomes active and phosphorylates their substrates on their serine and/or threonine sites in different signaling microdomains, leading to the conduction of cellular biological function **(B)** In the presence of cAMP, the regulatory and catalytic subunits that comprise the PKA holoenzyme dissociate. Then protein kinase inhibitor peptide (PKI) inhibits the activity of PKA by binding to the free C subunit of PKA and inhibiting the phosphorylation of PKA substrates.

PKA is inactive in the absence of the triggering cAMP. Like most protein kinases, PKA was found in an inactive state under basal conditions and activated by a variety of ligand-induced regulatory mechanisms (Taylor et al., 2012b). The underlying molecular mechanism for the activation of PKA by cAMP intracellularly has been extensively studied and is the prototype of protein kinase activation. PKA is a tetrameric holoenzyme comprising two regulatory (R) and two catalytic (C) subunits (Taylor et al., 1990). The C subunit has three isoforms Cα, Cβ, and Cγ (Kirschner et al., 2009; Taylor et al., 2012a), while the R subunit can be categorized into two subtypes (type I and II) according to their distribution, biochemical properties (e.g., sensitivities to cAMP), and affinity to A-kinase Anchoring Protein (AKAP). Each subtype of R subunits includes α and β isoforms. The R subunits serve three roles: binding to and inhibiting the C subunit and anchoring PKA to scaffolding protein AKAP. The RI subunit has a pseudosubstrate sequence, which has high affinity with the C subunit but cannot be phosphorylated by the C subunit, thus inhibiting the C subunit. The RII subunit is a substrate and also an inhibitor of the C subunit though phosphorylated RII does not dissociate from the C subunit in the absence of cAMP. In general, most AKAPs show more specificity to the RII subunit than to the RI subunit though D-AKAP1 and D-AKAP2 may have the same high affinity to RI and RII. RI has been found in cytosol and mitochondria. Currently more than 70 AKAPs have been identified (Zhu et al., 2019). AKAP-mediated targeting of PKA to certain subcellular compartments provides the spatial and temporal regulation of the whole signaling cascade (Lin et al., 1998; Torres-Quesada et al., 2017). In the presence of cAMP, each regulatory subunit binds to two molecules of cAMP at separate allosteric binding sites (one high affinity site and one low affinity site), leading to reduced affinity between the R and the C subunits and separation of the holoenzyme of PKA into a regulatory subunit dimer and two monomeric catalytic subunits. The released catalytic subunits become active and phosphorylate their substrates on serine and/or threonine sites in different signaling microdomains, leading to the conduction of cellular biological function (Figure 1A; Dalton and Dewey, 2006).

PKA signaling orchestrated by AKAP plays critical roles in regulating cardiovascular (Zhu et al., 2019), neuronal (Dell’acqua et al., 2006), and other functions and diseases (Reggi and Diviani, 2017). In the brain, an AKAP called Yotiao is anchored to the plasma membrane of neurons and scaffolds PKA and the NR1 subunit of post-synaptic N-methyl-D-aspartic acid (NMDA) receptor (Feliciello et al., 1999). When PKA is activated, it causes substantial phosphorylation and activation of NMDA, which plays a vital role in the regulation of synaptic plasticity (Feliciello et al., 1999; Westphal et al., 1999). AKAP79/150 may anchor the L-type Ca^2+^ channels to the membrane in a supermolecular complex to coordinate the regulation of the channel (Pallien and Klussmann, 2020). WAVE1 (Wiskott-Alrich syndrome protein family verprolin homology protein 1), an AKAP, is associated with cytoskeleton to transduce and regulate basic cellular functions (Scott, 2003). Muscle specific AKAP (mAKAP) on the nuclear envelope may regulate PKA activity, which is critical for gene expression in the nucleus (Rababa’h et al., 2014). AKAPs on the outer membrane of mitochondria regulate PKA-dependent phosphorylation of Bad to exert an antiapoptotic effect (Ould Amer and Hebert-Chatelain, 2018). One research group suggested that the released free PKA C subunits do not diffuse far away from the microdomains (Smith et al., 2013, 2017) but this has been challenged (Walker-Gray et al., 2017). Nonetheless, activated PKA C has been shown to diffuse into the nucleus (Martin et al., 2007) to phosphorylate nuclear proteins including transcription factors such as the cAMP-response element binding (CREB) protein (Dalton and Dewey, 2006; Roskoski, 2015).

## Protein Kinase Inhibitors as an Endogenous PKA Regulatory Element and a Tool for PKA Inhibition

The termination of PKA signaling includes multiple counteracting mechanisms: the degradation of cAMP by phosphodiesterases (PDEs; London et al., 2020), the dephosphorylation of PKA substrates by phosphatases (Burdyga et al., 2018), and the inhibition of PKA by PKI, a soluble peptide in the cell (Meinkoth et al., 1993). While many studies have addressed the degradation of cAMP and the dephosphorylation of PKA substrates, the physiological and pathological roles of PKA inhibition by PKI remain to be elucidated. We will focus on the regulation of PKA by PKI in this review.

PKI is a heat-stable interfering peptide, firstly identified in skeletal muscle extract and which inhibited the activation of PKA (Posner et al., 1964, 1965). It has been demonstrated that PKI led to blocks the ability of cAMP to catalyze PKA activation (Walsh et al., 1971). PKI is widely distributed in a variety of tissues including the brain, heart, liver, testes, skeletal muscles, and pancreas, and thus plays an important role in the pathogenesis and progression of cancer, neurodegeneration disorders, and drug addiction (Nestler, 1997; Farrow et al., 2003; Sun et al., 2005; Dalton and Dewey, 2006). There are three endogenous PKI isoforms, PKIα, β, and γ, which expressed in cell-specific and/or tissue-specific expression patterns. In the cell, it seems that all three isoforms are concentrated in the nucleus and at high level in the cytosol (Dalton and Dewey, 2006). PKIα is highly expressed in skeletal and cardiac muscles, brain, liver, pancreas, and expressed moderately in kidney, and colon; while PKIβ is highly expressed in the testis and moderately expressed in the spleen and cerebellum (Van Patten et al., 1992). PKIγ is highly expressed in the heart and moderately expressed in the brain, pancreas, lungs, liver, skeletal muscle, kidney, spleen, prostate, testis, ovary, small intestine, and colon (Zheng et al., 2000). In this review, we mainly focus on the mechanisms of PKI inhibiting PKA activation and the method of PKA inhibition taking advantage of PKI.

## The Structure of PKI

All three PKI isoforms contain a PKA inhibition domain near the N-terminus and a nuclear export signal (NES) domain (Dalton and Dewey, 2006). Small synthetic peptide analogs studies have revealed that the main structural determinant of PKI inhibitory domain is composed of amino acid residues, which play a role in PKI inhibitory activity. The amino acid sequences in the inhibitory domain of PKI are similar to the regulatory subunit of PKA that allow them to bind to the PKA catalytic subunit and inhibit its activity (Dalton and Dewey, 2006). In general, PKI does not have many α-helix and β-structures but contains many random coil and turn structures. This gives rise to in many conformations and allows the protein to easily refold when temperature decreases after heating.

PKIα consists of 75 amino acids, of which amino acids 1–25 are the PKA inhibition domain (Ki = 0.2 nM) and the NES is within residues 37–46 (LALKLAGLDI). This NES contains several hydrophobic amino acids and is one of the strongest NES identified (Dalton and Dewey, 2006). Normally this NES is masked and can be exposed only when PKI binds to PKA C subunit. PKIβ is a peptide of 70 amino acids sharing 41% homology with PKIα, mostly in the PKA inhibition domain and the NES domain. The PKA inhibition domain of PKIβ is almost the same as the inhibition domain of PKIα except it does not have tyrosine 7, rendering a weaker inhibition (Ki = 7.1 nM). PKIγ is composed of 75 amino acids and shares the homology in the PKA inhibition and NES domains, results a 30% homology with PKIα. PKIγ has a unique amino acid, cysteine 13, in the PKA inhibition domain, which is believed to play a role in its high affinity to PKA C subunit (Ki = 0.4 nM; Dalton and Dewey, 2006).

## The Regulation of PKA Activity by PKI

PKI regulates the activation of PKA *via* two mechanisms: direct inhibition of PKA and the regulation of intracellular localization of PKA. The inhibition of PKA activation relies on the pseudosubstrate amino acid sequence in PKI located at the N-terminus, which is similar to that in the RI subunit of PKA. PKI binds to the catalytic subunit of PKA with high affinity *via* the pseudosubstrate sequence but cannot be phosphorylated, leading to the inhibition of PKA activity (Figure 1B; Ashby and Walsh, 1972; Demaille et al., 1977). In this process, PKI acts as a potent competitive inhibitor for active PKA catalytic subunit (Kemp et al., 1977; Hofmann, 1980). PKI binding to PKA C subunit, which is a process depending on intracellular divalent ion concentration and ATP, leads to a closed, enzymatically inactive form of PKA C (Zimmermann et al., 2008). The PKI-mediated inhibition of free catalytic subunits of PKA only occurs following the cAMP-mediated dissociation of the regulatory and catalytic subunits since PKI lacks a special binding site for cAMP presenting on the regulatory subunits (Dalton and Dewey, 2006).

## The Regulation of Nuclear PKA Activity by PKI

**Figure 2:**
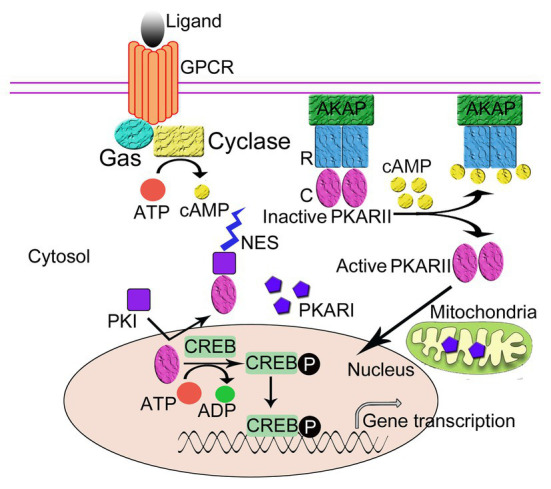
The regulation of the intracellular localization of PKA. The regulatory subunits (R) of PKA bind to A-Kinase Anchoring Proteins (AKAPs), which anchor the PKA holoenzyme to the plasma membrane thus regulates the intracellular localization of PKA. Protein kinase inhibitor peptide (PKI) also regulates the intracellular localization of PKA. Following activation by cAMP, the R subunits of PKA dissociate from the catalytic subunits (C). The C subunits diffuse into cell nucleus and phosphorylate the cAMP-response element binding protein (CREB), which can then activate gene transcription. PKI enters the nucleus and binds to the free C subunits of PKA and exposes the nuclear export signal (NES) of PKI to transport the C subunit out of the nucleus in an ATP-dependent manner thus inhibits the activity of C subunits.

PKA plays an important role in the regulation of gene expression by phosphorylating transcription factors in the nucleus, and PKI functions as an important regulator of nuclear PKA activity. Free nuclear PKA catalytic subunits can be either from the diffusion of free PKA catalytic subunits into the nucleus (Gonzalez and Montminy, 1989; Harootunian et al., 1993; Martin et al., 2007) or from the dissociation of PKA holoenzyme anchored in the nucleus by nuclear AKAP, e.g., AKAP95 (Clister et al., 2019). In the nucleus, the free catalytic subunits catalyze the phosphorylation of some nuclear proteins, such as the transcription factors CREB and CREM. Phosphorylated CREB then binds to cAMP responsive element (CRE) promoters and activates gene transcription (Figure 2; Habener, 1990; Brindle and Montminy, 1992).

PKI, a small soluble peptide, can diffuse into the nucleus from the cytosol through the nuclear pore to bind to and inhibit the activity of the free catalytic subunits of PKA in the nucleus. The binding of PKI to the PKA catalytic subunit exposes the NES of PKI, which enables PKI to transport the catalytic subunits of PKA out of the nucleus to the cytoplasm in an ATP-dependent manner (Fantozzi et al., 1994; Wiley et al., 1999). Recently, it has been proposed that PKA C subunit goes through a multi-state recognition pathway for PKI. First, PKA C recognizes a most competent conformation of PKI, and then PKI binds to the binding pocket and slowly rearranges its conformation, making the PKI region more structured and increasing the helical content of the region N-terminal to the NES. Subsequently, NES recruits CRM1/RanGTP complex for nuclear export (Olivieri et al., 2020). In the cytoplasm, the free catalytic subunits of PKA are able to reassociate with the regulatory subunits to form the cAMP-dependent protein kinase holoenzyme and restore cAMP regulation to the cell (Dalton and Dewey, 2006). Through this process, PKI can function to regulate the amount of free PKA C in the nucleus. It should be noted that in this regulatory process, two domains of PKI with unique functions are involved, namely the inhibitory domain near the NH_2_-terminus and the NES domain located between the NH_2_-terminus and the COOH-terminus, where the former allows for the realization of the inhibition of the cAMP-dependent protein kinase inhibition and the latter mediates the nuclear export of the catalytic subunits of the cAMP-dependent protein kinase (Henderson and Eleftheriou, 2000; Dalton and Dewey, 2006).

## Experimental Methods for PKA Inhibition

As discussed above, PKA mediates the signals from the extracellular side to the intracellular side and various kinds of life processes demand to termination and/or reverse phosphorylation of proteins catalyzed by PKA (Akabane et al., 2016; Bachmann et al., 2016; Tao et al., 2016; Yamauchi et al., 2016). So far, several kinds of methods have been developed for the inhibition of PKA in research, including the development of small molecules such as H89, and the construction of synthetic peptide analogs of PKI as well as the creation of PKA specific inhibition animal models taking advantage of PKI (Glass et al., 1995; Lochner and Moolman, 2006; Inoue et al., 2013; Zhang et al., 2013a; Zynda et al., 2014). Although in this review, we mainly aim to discuss the related knowledge of PKI, we also introduce widely used small molecular compounds, Rp-cAMP, KT-5720 and H89, to facilitate the subsequent discussion of PKI.

## Non-Peptide PKA Inhibitors

There are multiple chemical PKA inhibitors including cAMP analogs, H8 and its derivatives and KT5720. Rp-cAMP is a commonly used cAMP analog that competitively binds to the cAMP binding sites on the PKA R subunit to prevent PKA activation (De Wit et al., 1984). Rp-cAMP can be hydrolyzed by intracellular PDEs and thus PDE resistant modifications, for example, Rp-8-Br-cAMP, have been added on to the molecule. The effectiveness of Rp-cAMP and its derivatives depends on the intracellular cAMP concentration, which often requires high concentration (tens of micromolar) in the cell to effectively inhibit PKA activation. As a cAMP analog, it also binds to other cAMP targets such as nucleotide-gated channels and EPAC (exchange protein activated by cAMP; Murray, 2008). It has been shown that Rp-8-Br-cAMP can be more specific for PKA than EPAC and thus it was used to distinguish the antidepressant effects of PKA and EPAC (Liebenberg et al., 2011). It was also used to reveal the molecular mechanism of norepinephrine on natural killer cells’ cytotoxicity (Sun et al., 2018).

H89 and KT5720 exert their PKA inhibition effect *via* a common mechanism, binding to the ATP binding pocket of PKA C subunit. As such, their effectiveness depends on intracellular ATP concentration. KT5720 is a compound from the fungus *Nocardiopsis* sp., having a Ki of 60 nM for PKA inhibition. It is relatively non-specific and inhibits multiple protein kinases, such as PKB, MESK, GSK-3βα, and AMPK, almost as effectively as on PKA (Murray, 2008). Additionally, it inhibits overall transcription and enhances the affinity of acetylcholine for M1 receptors (Lochner and Moolman, 2006).

H89, N-[2-(pbromocinnamylamino)ethyl]-5-isoquinoline sulfonamide, is a cell permeable, relatively PKA-specific derivative from its predecessor, H8, a PKA, and protein kinase G (PKG) inhibitor. It is by far the most widely used PKI inhibitor in research related to PKA (Wright and McCarthy, 2009). This is evident from a search in the PubMed database with H89 as an approach of PKA inhibition, which identified over 1,000 studies from the year of 2010 to 2020. H89 has been extensively regarded as a selective and potent PKA inhibitor with the inhibition constant (Ki) of 0.048 μM for PKA, and 10 times higher Ki for PKG (Chijiwa et al., 1990). The mechanism underlying the inhibitory effect of H89 is that it functions as a robust and effective competitive antagonist of ATP at the binding site on the PKA catalytic subunit (Engh et al., 1996; Murray, 2008). As discussed above, it is necessary for the catalytic monomer to bind to ATP in order to function as a phosphorylating enzyme for serine or threonine residues on specific substrates. As a result, the competitive blockade of the binding of PKA catalytic subunit to ATP effectively undermines the original phosphorylating effect of PKA, thus inhibiting PKA functions. Numerous studies have reported the effects of H89 on different cellular processes or diseases. For example, it was demonstrated by Zhang et al. (Zhang et al., 2016) that the application of H89 in the concentration of 4 μM significantly enhanced the survival and clonogenicity of dissociated human embryonic stem cells (hESCs) without influencing their pluripotency. They reported that H89 could inhibit the dissociation-induced phosphorylation of PKA and its two substrates [Rho-associated coiled-coil containing protein kinase (ROCK), and myosin light chain (MLC2) and myosin phosphatase target subunit 1 (MYPT1)]. This study indicated H89 might be beneficial in transplantation. Furthermore, it was demonstrated recently that H89 attenuated synaptic dysfunction and neuronal cell death following ischemic injury through inhibition of PKA and a subsequent decrease in apoptosis, indicating that H89 might be used for brain recovery after ischemic stroke (Song et al., 2015). In addition, several other studies have demonstrated that H89 could function as an autophagy inducer and inflammatory inhibitor, indicating the potential application of H89 in the treatment of many kinds of diseases in clinic (Gomez-Concha et al., 2011; Reber et al., 2012; Inoue et al., 2013).

Despite the mechanistic insight provide by studies utilizing H89, those inhibitors are so far cannot be applied without cautions (Murray, 2008). Firstly, the IC_50_ of H89, the concentration at which a compound inhibits 50% of a given activity, varies according to the ATP concentration, which makes it hard for researchers to get the effective dose of H89 in the treatment of cells. Secondly and more importantly, H89 is a nonspecific inhibitor with effects on ion channels, receptors, Ca^2+^-ATPase, small GTPase RhoA, and other kinases such as ROCK1 and PKB (Lochner and Moolman, 2006; Murray, 2008). This largely limits the use of H89 in the study of PKA.

Taken together, non-peptide PKA inhibitors suffer from the drawbacks of high concentration needed (e.g., Rp-cAMP and H89) or many non-specific effects off the target PKA. Consequently, more effective and specific inhibitors for PKA are required for the study of PKA.

## Synthetic Peptide Analogs of PKI

As described above, PKI is an endogenous molecule that specifically inhibits PKA activity with a low IC_50_, thus PKI may provide a more effective and specific approach in the inhibition of PKA. Since the PKA inhibition domain has been identified, several synthetic peptide forms of PKI have been developed and widely applied for the study of PKA function. So far, synthetic peptide analogs of PKI include PKI-(6-22)-amide (Thr-Tyr-Ala-Asp-Phe-Ile-Ala-Ser-Gly-Arg-Thr-Gly-Arg-Arg-Asn-Ala-Ile-NH2), PKI-(14-24)-amide (Gly-Arg-ThrGly-Arg-Arg-Asn-Ala-Ile-His-Asp-NH2), andPKI-(5-24)-amide (Thr-Thr-Tyr-Ala-Asp-PheIle-Ala-Ser-Gly-Arg-Thr-Gly-Arg-Arg-Asn-Ala-Ile-His-Asp-NH2; De Boer et al., 2005; Guergnon et al., 2006; Khan and Maitra, 2013). Using electrospray ionization mass spectrometry (ESI-MS), Boer et al. (De Boer et al., 2005) determined the inhibition potency of these three PKI peptides and demonstrated that PKI-(6-22)-amide had the highest inhibition potency followed by PKI-(5-24)-amide and then PKI-(14-24)-amide. This conclusion was drawn according to the Ki calculated (7.4, 19, and 340 nM, respectively) and IC_50_ determined (8.4, 22, and 380 nM, respectively).

Synthetic PKA inhibiting segments of PKI have been used to stabilize thPKA C subunit to reveal its 3D structure (Chu et al., 2017). To be used for studies with live cells, PKI has been either modified to make it permeable to the cell membrane or cloned into viral vectors. TAT-capping (Matsushita et al., 2001), myristoylation (Harris et al., 1997; Hodges et al., 1997), and most recently hydrocarbon-stapled (Manschwetus et al., 2019) modifications have been added to PKI to make it cell permeable. A light activatable PKI has been developed for switching on PKA inhibition in live cells (Yi et al., 2014). PKI as a specific PKA inhibitor has been used in cultured cell studies in many experiments. For example, a recent study showed that inhibiting PKA by PKI can divert G protein coupled receptor (GPCR) signals toward cell growth in cancer cells (Hoy et al., 2020). It was recently demonstrated that the inhibition of PKA by PKI-(6-22)-amide significantly reduced the toxicity of Taxol and Taxane to prostate cancer cells, indicating that PKA inhibition could influence the response of cancer cells to Taxol and Taxane-based therapy (Zynda et al., 2014). Maitra et al. (2014) reported that the inhibition of PKA with PKI-(6-22)-amide in combination of H89 could largely induce insulin-stimulated germinal vesicle breakdown (GVBD) and mitogen-activated protein kinase (MAPK)-mediated signaling pathway in full-grown oocytes in zebrafish, thus the maturation of zebrafish oocytes. PKI-(6-22)-amide alone was sufficient to resume GVBD and MAPK activation in intact perch oocytes and induced the oocyte maturation in *Anabas testudineus* (Khan and Maitra, 2013). Besides, PKI-(6-22)-amide was demonstrated to influence the stimulation of endothelial nitric-oxide synthase (eNOS) activity by bone morphogenetic protein receptor II (BMPRII) and the level of induced Ca^2+^ (Perez-Cornejo and Arreola, 2004; Liu et al., 2008, 2009; Gangopahyay et al., 2011). In the central nervous system, PKA inhibition by PKI-(6-22)-amide and PKI-(Myr-14-22)-amide could reverse the low-level morphine antinociceptive tolerance in mice (Dalton et al., 2005). PKI-(6-22)-amide can also significantly inhibit the spontaneous release of glycine and subsequent postsynaptic currents in neurons (Katsurabayashi et al., 2004). PKI-(6-22)-amide has been used to demonstrate a role for in cancers, endocrine disorders, and cardiovascular. For example, PKI-(6-22)-amide can revert the proliferative effect of oxytocin-treated MDA-MB231 breast-carcinoma cells *via* inhibiting cAMP-PKA pathway (Cassoni et al., 1997). In cultured islets exposed to 5.5 mmol L of glucose, PKI 6-22 amide did not inhibit glucose-induced insulin secretion, but it can inhibit amplification stimulated by the adenylyl cyclase activator, forskolin (Thams et al., 2005).

Recently, a novel animal model with *in vivo* cardiac specific PKA inhibition taking advantage of PKI was reported by our group (Zhang et al., 2013a, 2019). A PKI transgenic mouse model as generated by overexpressing the PKA inhibition domain the coding sequence for amino acids 1–25 (MTDVETTYADFIASGRRNAIHD) of mouse PKIα (mouse Entrez gene) fused with GFP in a cardiac specific and inducible manner (Gossen et al., 1994; Sanbe et al., 2003). Amino acids 1–25 of mouse PKIα have the PKA inhibitory domain but not the NES (Chen et al., 2002; Tang et al., 2010; Zhang et al., 2013a). We demonstrated that PKA activity can be inhibited by as much as 90% in the crude extract from double transgenic (containing both PKI-GFP and ttA transgenes) hearts overexpressing a fusion gene containing the nucleotide sequence coding the amino acids 1–25 of PKIα and GFP (PKI-GFP), compared with control hearts (Zhang et al., 2013a). An adenovirus containing the fusion gene (AdPKI-GFP) was also created to infect cultured adult feline ventricular myocytes. We showed that PKA inhibition provides cardiac protection in chronic exposure of the heart to β-adrenergic agonists. Furthermore, using AdPKI-GFP as a tool to inhibit PKA activation, we unveiled another novel cAMP-dependent cardioprotective pathway, the EPAC/Rac1/ERK signaling pathway.

Despite the fact that synthetic peptide analogs of PKI are likely to be a more specific inhibitor of PKA compared with other compound inhibitors like H89, they have their own issues. High concentrations of synthetic peptide analogs of PKI also inhibits PKG, thus caution still needs to be applied when using PKI analogs to selectively inhibit PKI (Glass et al., 1992).

## Other Biological Ways of PKA Inhibition

Although synthetic peptide analogs of PKI are more effective and specific compared with H89, they have their drawback as an imperfect PKA inhibitor. With the development of molecular biology techniques, novel methods for inhibiting or knocking down PKA have been developed, which has facilitated our understanding of PKA’s role in biological and pathological processes.

For cellular studies, several methods are available for reducing or eliminating PKA activity. Among them, the most popular is small interfering RNAs (siRNA) or RNA interference (RNAi). Since siRNA was reported in 1998 (Fire et al., 1998), it has been used for knocking down the expression of various kinds of proteins. For PKA, siRNA was used to knockdown at least two PKA isoforms, namely α and β isoforms (Dumaz and Marais, 2003; Rudolph et al., 2007; Monaghan et al., 2008; Murray, 2008). Recently, this approach has been proven to be effective in several kinds of cells including human smooth muscle cells, cardiomyocytes, preadipocytes, adipocytes as well as retinal endothelial cells (Peng et al., 2012; Toneatto et al., 2013; Zhang and Steinle, 2013; Zhang et al., 2013b; Kang et al., 2014). The expression of cardiac PKA could be effectively knocked down (>70%) through intramyocardial injection of PKA-siRNAs (Zhang et al., 2013b). Besides PKA siRNA, the introduction of a nonfunctioning PKA mutant, such as a dominant negative forms of PKA, has been used for specific suppression PKA in the examination of the function of PKA (Howe and Juliano, 2000; Hayashida et al., 2006).

## Conclusion

PKA has been extensively studied, and it is an important regulator of various cellular processes and disorders in almost all systems. Recently, methods taking advantage of PKI have proven to be more effective and specific in the inhibition of PKA than chemical PKA inhibitors and widely used in studies regarding biological roles of PKA. However, PKI can inhibit PKG at high concentrations and thus should be used with caution. More research on PKI ought to be conducted to improve its specificity.

## Author Contributions

CL, PK, and XZ were in charge of searching all the relative papers and writing this manuscript. JZ was in charge of drawing the picture. XC guided the organization and drafting of this manuscript. All authors contributed to the article and approved the submitted version.

### Conflict of Interest

The authors declare that the research was conducted in the absence of any commercial or financial relationships that could be construed as a potential conflict of interest.
